# Mutation analysis of *SLC26A4* (Pendrin) gene in a Brazilian sample of hearing-impaired subjects

**DOI:** 10.1186/s12881-018-0585-x

**Published:** 2018-05-08

**Authors:** Renata Watanabe Nonose, Karina Lezirovitz, Maria Teresa Balester de Mello Auricchio, Ana Carla Batissoco, Guilherme Lopes Yamamoto, Regina Célia Mingroni-Netto

**Affiliations:** 1 Centro de Pesquisas sobre o Genoma Humano e Células-Tronco, Departamento de Genética e Biologia Evolutiva, Instituto de Biociências, Universidade de São Paulo, São Paulo, Brazil; 2 Laboratório de Investigação Médica/LIM32 do Hospital das Clínicas da Faculdade de Medicina da Universidade de São Paulo, São Paulo, Brazil

**Keywords:** Hereditary deafness, *SLC26A4*, Pendrin gene, Brazil

## Abstract

**Background:**

Mutations in the *SLC26A4* gene are associated with Pendred syndrome and autosomal recessive non-syndromic deafness (DFNB4). Both disorders have similar audiologic characteristics: bilateral hearing loss, often severe or profound, which may be associated with abnormalities of the inner ear, such as dilatation of the vestibular aqueduct or Mondini dysplasia. But, in Pendred syndrome (OMIM #274600), with autosomal recessive inheritance, besides congenital sensorineural deafness, goiter or thyroid dysfunctions are frequently present. The aim of this study was to determine whether mutations in *SLC26A4* are a frequent cause of hereditary deafness in Brazilian patients.

**Methods:**

Microsatellite haplotypes linked to *SLC26A4* were investigated in 68 families presenting autosomal recessive non-syndromic deafness. In the probands of the 16 families presenting segregation consistent with linkage to *SLC26A4*, Sanger sequencing of the 20 coding exons was performed. In an additional sample of 15 individuals with suspected Pendred syndrome, because of the presence of hypothyroidism or cochleovestibular malformations, the *SLC26A4* gene coding region was also sequenced.

**Results:**

In two of the 16 families with indication of linkage to *SLC26A4*, the probands were found to be compound heterozygotes for probably pathogenic different mutations: three novel (c.1003 T > G (p. F335 V), c.1553G > A (p.W518X), c.2235 + 2 T > C (IVS19 + 2 T > C), and one already described, c.84C > A (p.S28R). Two of the 15 individuals with suspected Pendred syndrome because of hypothyreoidism or cochleovestibular malformations were monoallelic for likely pathogenic mutations: a splice mutation (IVS7 + 2 T > C) and the previously described c.1246A > C (p.T416P). Pathogenic copy number variations were excluded in the monoallelic cases and in those with normal results after Sanger sequencing. Additional mutations in the *SLC26A4* gene or other definite molecular cause for deafness were not identified in the monoallelic patients, after exome sequencing.

**Conclusions:**

Biallelic pathogenic mutations in *SLC26A4* explained ~ 3% of cases selected because of autosomal recessive deafness. Monoallelic mutations were present in ~ 13% of isolated cases of deafness with cochleovestibular malformations or suspected Pendred syndrome. These data reinforce the importance of mutation screening of *SLC26A4* in Brazilian subjects and highlight the elevated frequency of monoallelic patients.

## Background

Hearing loss may be explained by genetic, environmental or multifactorial causes. Hereditary hearing loss is considered one of the most striking examples of genetic heterogeneity, since it might exhibit all Mendelian inheritance patterns, as well as mitochondrial inheritance. Near 70% of individuals with hereditary hearing loss are referred as non-syndromic and, in remaining 30%, additional clinical features are present, characterizing syndromic hearing loss. Autosomal recessive inheritance contributes to 80% of nonsyndromic hereditary hearing loss, and mutations in the gene encoding the gap junction protein Connexin 26 (*GJB2* – DFNB1) are present in about 50% of the recessive cases; autosomal dominant inheritance is observed in 10–20% of the cases, and X-linked inheritance in 2–3%. The frequency of mitochondrial mutations is about 1% [1–3].

Mutations in the *SLC26A4* gene (NM_000441) were found to be causative of two autosomal recessive disorders, Pendred syndrome (OMIM # 274600) and one of the forms of non-syndromic autosomal recessive hearing loss (DFNB4; #600791) [4]. Pendred syndrome (PS) is characterized by bilateral sensorineural hearing loss, commonly severe to profound with prelingual onset, vestibular dysfunction, cochleovestibular malformations, such as Mondini dysplasia and euthyroid goiter, and with onset in late childhood to early adulthood. The observed variability of these clinical features is frequently substantial, even within the same family. DFNB4 exhibits similar clinical features, except that the most common temporal bone abnormality is the enlarged vestibular aqueduct (EVA), and thyroid defects are not observed [5]. Because of the variable expressivity and overlap of the clinical features, the two conditions may be considered as subsets of the spectrum of clinical manifestations of one single genetic entity [5].

The *SLC26A4* gene encodes pendrin, a protein with 780 amino acids that belongs to the SLC26 anion transporter family [4, 6]. The human pendrin is generally expressed in the inner ear, mainly in endolymphatic sac and hair cells, and in the follicular cells of the thyroid [6–9]. Impaired function of pendrin was associated with endolymph acidification, leading to auditory sensory transduction defects. It is believed that its function in normal inner ear is related to Ca2+ re-absorption inhibition and bicarbonate/chloride exchanging, playing a role in pH homeostasis [8, 9]. In the thyroid, iodide efflux from cells to follicular lumen is allowed by pendrin functioning as an electroneutral iodide/chloride exchanger [6, 7].

Mutations in the *SLC26A4* gene are reported to be the most frequent cause of hereditary hearing loss in East Asia, and the second most common cause worldwide, after Connexin 26 (*GJB2*) gene mutations [10–16]. The purpose of our study was to investigate the contribution of *SLC26A4* mutations to hearing loss in Brazilian patients.

## Methods

### Patients

A total of 31 unrelated patients were selected to the molecular study of the *SLC26A4* gene by Sanger sequencing, divided into two groups, based on clinical and genetic findings, as follows: (A) A sample of 68 pedigrees with hearing loss with presumed autosomal recessive inheritance (pedigrees with at least two affected sibs, born to hearing parents, or with at least one affected individual born to consanguineous parents, regardless of clinical data) was considered eligible to haplotype analysis with microsatellite (STR) markers linked to the DFNB4. The haplotype analysis with microsatellite (STR) markers allowed exclusion of segregation with DFNB4 in 52 pedigrees. Thus, 16 probands from the remaining pedigrees with haplotype segregation compatible with DFNB4 were selected to sequencing of the *SLC26A4* gene. (B) 15 patients suspected of Pendred syndrome, because of the presence of hearing loss associated with thyroid dysfunction, or because of the presence of hearing loss with EVA or presenting hearing loss associated to other cochlear/vestibular malformation. In this group, 6 had hearing loss and EVA, 5 had hearing loss and Mondini dysplasia, and 4 had hearing loss and hypothyroidism. Most patients in this group were isolated cases.

Mutations in *GJB2*, two deletions near *GJB6* gene [Δ (*GJB*6–D13S1830) and Δ(*GJB*6–D13S1854)], and the mitochondrial m.1555A > G mutation, had been previously excluded as the genetic cause of hearing loss in all probands, from both groups.

### Molecular analysis

Blood samples were collected after written informed consent was obtained from all individuals or their legal guardians (if patients were under 18). The project was approved by the Ethics Committee, Instituto de Biociências, Universidade de São Paulo (Protocol n^o^ 109/2010). DNA was extracted by standard procedures using phenol/chloroform or using Autopure LS (Gentra Systems, Minneapolis, MN, USA).

### STR genotyping

Microsatellite STR markers on 7q31, linked to the DFNB4 locus, were genotyped and used to perform haplotype analysis (D7S2420, D7S496, D7S2459, D7S2456, D7S799). One of the microsatellites, D7S2459, maps to intron 10 of the *SLC26A4* gene. Primer sequences for the STR amplification were obtained from databases (http://genome.ucsc.edu and http://www.ncbi.nlm.nih.gov), except for the D7S799 marker, for which the reverse primer was designed using PRIMER 3 [17] (http://bioinfo.ut.ee/primer3-0.4.0/). The polymorphic fragments were analyzed using the GeneMapper software following capillary electrophoresis in the ABI 3730 DNA Analyzer (Applied Biosystems, Carlsbad, CA, USA).

### Sanger sequencing of *SLC26A4* coding region

PCR amplification of the 20 coding exons of *SLC26A4* (2–21) and their flanking intronic sequences was performed using primers already described in Everett et al. [4], Scott et al. [18] and Lofrano-Porto et al. [19], except for exons 11 and 12, 15, 17 and 21, which had the primers designed using “Primer3” software. The PCR fragments were sequenced using the ABI BigDye Terminator v3.1 Cycle Sequencing Kit and the ABI 3730 DNA Analyzer (Applied Biosystems, Carlsbad, CA, USA).

### MLPA analysis

When only one pathogenic variant was detected (two patients) or none possibly pathogenic allele was identified after Sanger sequencing (27 patients), samples were submitted to MLPA analysis, searching for possible copy number variations (CNVs) encompassing the *SLC26A4* gene. MLPA was performed using the SALSA MLPA KIT P280-B1 Pendred-*SLC26A4* kit (MRC Holland, Amsterdam, Netherlands), following the manufacturer’s instructions. The amplification products were subjected to capillary electrophoresis using the ABI 3730 DNA Analyzer (Applied Biosystems, Carlsbad, CA, USA). The results were analyzed using the Gene Marker software (https://softgenetics.com/GeneMarker.php).

### Massive parallel sequencing and bioinformatic analysis

Massive parallel sequencing of the whole exome was performed in two samples, from individuals with only one detected likely pathogenic variant in the *SLC26A4* gene (monoallelic). DNA samples were submitted to whole-exome sequencing at Laboratório Central de Tecnologias de Alto Desempenho em Ciências da Vida (LaCTAD) (University of Campinas, SP, BR). Sample libraries were prepared using the ‘TruSeq Custom DNA Library Preparation Kit’ from Illumina (Illumina INC, San Diego, California, USA). Whole exome was captured with Illumina’s ‘TruSeq Expanded Exome Enrichment Kit’ (target regions of 62 Mb). Illumina HiSeq 2500 was used to sequence the samples with paired-end fragments of 100 × 100 and average coverage of 120×. Alignment of fastq files to human reference hg19 was done with Burrows-Wheeler Aligner (BWA) [20], generating SAM files. SAM to BAM conversion, BAM files sorting and PCR duplicates marking were executed with Picard (http://broadinstitute.github.io/picard/). Genome Analysis Tool Kit (GATK) [21] was used in the steps of BAM processing (realignment based upon known local indels and variant quality score recalibration), and variant call (with Unified Genotyper). Variants in the VCF file were annotated with Annovar [22]. Variant frequencies were compared to the 1000 genomes (http://www.1000genomes.org), ESP6500 (http://evs.gs.washington.edu/EVS/), 65000 exomes from Exome Aggregation Consortium (ExAC) (http://exac.broadinstitute.org/) databanks and ABraOM (http://abraom.ib.usp.br/) [23]. The Deafness Variation Database (http://deafnessvariationdatabase.org) [24] and ClinVar were also consulted (https://www.ncbi.nlm.nih.gov/clinvar/). PolyPhen2 [25] SIFT [26] and Mutation Taster [27] were used for in silico pathogenicity prediction of the mutations. To address the effect of the splice site mutations, two softwares were used: NNSPLICE 0.9 version (http://www.fruitfly.org/seq_tools/splice.html) and NetGene 2 Server (http://www.cbs.dtu.dk/services/NetGene2/).

## Results

In order to determine whether mutations in the *SLC26A4* gene are a frequent cause of hereditary deafness in Brazilian patients, we analyzed 68 families presenting autosomal recessive non-syndromic hearing loss. Microsatellite haplotypes linked to the *SLC26A4* gene were investigated, and segregation was consistent with linkage to this gene in 16 families; linkage was excluded in the remaining 52 pedigrees. Sanger sequencing of the 20 coding exons was performed in samples from the 16 probands, as well as in 15 patients with suspected PS and/or presenting EVA or other cochlear-vestibular malformations. Detected variants are listed in Table 1.

**Table 1:** Heterozygous Variants and their predicted consequences detected after Sanger in both samples (pedigrees with microsatellite segregation compatible with linkage to *SLC26A4* and individuals presenting cochlear-vestibular malformations)

Cohort	Patient	Nucleotide	Protein	Location	Deafness variation database	SIFT (score), PolyPhen2 (score), MutationTaster (score), *Splice Prediction Tools*	1000 g	ESP6500	Conclusion (According to ACMG criteria)	Genotype	Protein location^b^
Families with autosomal recessive inheritance	**6**	**c.1003 T > G** ^a^	**p.F335V** ^a^	**exon 9**	**–**	**Damaging (0.003), Probably damaging (0.99), Disease causing (0.99)**	**–**	**–**	**likely pathogenic**	**Compound heterozygosis**	**External loop**
		**c.1553G > A** ^a^	**p.W518X** ^a^	**exon 14**	**–**	**-, −, Disease causing (1)**	**–**	**–**	**pathogenic**		**C-terminal**
	7	c.15C > A	p.G5G	exon 2	benign	–	0.006	0.005	benign	–	–
		IVS10 + 35G > T	–	intron 10	benign	–	0.003	0.003	benign	–	–
	**24**	**c.84C > A**	**p.S28R**	**exon 2**	**Likely Pathogenic**	**Damaging (0.003), Possibly damaging (0.92), Polymorphism (0.79)**	**0**	**0**	**likely pathogenic**	**Compound heterozygosis**	**N-terminal**
		**IVS19 + 2 T > C** ^a^	**SS** ^a^	**intron 19**	**–**	**-, −, Disease causing (1),** ***splice donor site abolished***	**–**	**–**	**pathogenic**		**C-terminal**
	44	c.218A > G	p.E73G	exon 3	–	Tolerated (0.313), Benign (0.00), Disease causing (0.77)	–	–	benign	–	–
		IVS15-18 T > A	–	intron 15	benign	–	0.013	0.02	benign	–	–
	51	IVS15 + 76G > C	–	intron 15	benign	–	0.039	0	benign	–	–
		c.1826 T > G	p.V609G	exon 17	benign	Tolerated (0.54), Benign (0.00), Polymorphism (0.19)	0.04	0.049	benign	–	–
		c.2130C > T	p.D710D	exon 19	benign	–	0.019	0.023	benign	–	–
		c.2218G > A	p.G740S	exon 19	benign	Tolerated (0,091), Benign (0.00), Polymorphism (0.99)	0.015	0.017	benign	–	–
Cases of deafness with suspected PS and/or presenting EVA or other cochleovestibular malformation	**71**	**c.1246A > C**	**p.T416P**	**exon 10**	**Pathogenic**	**Damaging (0.00), Probably damaging (1.00), Disease causing (0.99)**	**0**	**0**	**likely pathogenic**	**Heterozygosis (monoallelic)**	TM10/Cytosolic Interface
	76	c.898A > C	p.I300L	exon 7	benign	Damaging (0.02), Probably damaging (0.97), Disease causing (0.99)	0.005	0.004	benign	–	–
	78	IVS8-143 T > C		intron 8	–	–	–	–	benign	–	–
	**83**	**IVS7 + 2 T > C**	**SS**	**intron 7**	**Pathogenic**	**-, −, Disease causing (1)**	**0**	**0**	**pathogenic**	**Heterozygosis (monoallelic)**	TM7
	85	IVS15-18 T > A	–	intron 18	benign	–	0.013	0.022	benign	–	–

In bold, variants considered as pathogenic or likely pathogenic

^a^ indicates variants reported for the first time in this study. ^b^According to Bassot et al. 2017 [52]

After variant filtering, according to classification in different databases and bioinformatics prediction of pathogenicity, six different variants were found in four probands that could be considered as probably causative of their autosomal recessive hearing loss. These variants were classified according to ACMG guidelines [28]. Half of them were missense variants (3/6, 50%); a novel nonsense mutation [c.1553G > A (p.W518X)] was identified; two splice site variants were detected, one novel (IVS19 + 2 T > C), and the other, previously described as pathogenic (IVS7 + 2 T > C).

**Fig. 1:**
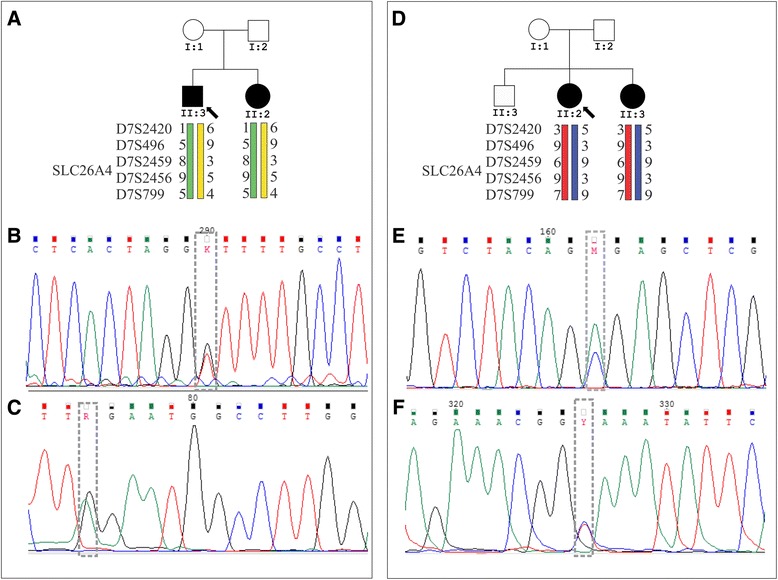
Pedigrees of families 6 (**a**) and 24 (**d**) showing the segregation of the pathogenic mutations. Chromatograms showing the probably pathogenic mutations found to segregate with deafness in family 6 (**b** and **c**) and family 24 (**e** and **f**)

Probands 6 and 24 (Table 1) are compound heterozygotes for two different likely pathogenic mutations: c.1003 T > G (p.F335 V) and c.1553G > A (p.W518X) in Patient 6; the variant c.84C > A (p.S28R) and the novel splice site mutation IVS19 + 2 T > C in Patient 24. Figure 1 shows the STR haplotypes and chromatograms of the mutations present in the two families.

It is noteworthy that in two probands (Patients 71 and 83), only one likely pathogenic mutation was detected and they were thus classified as “monoallelic” (Table 1). MLPA analysis performed in these two patients with only one detected pathogenic variant, and in those 27 patients who had no possibly pathogenic allele detected after Sanger sequencing, revealed no *SLC26A4* deletion or duplication.

The DNA samples of the two monoallelic patients were submitted to whole exome sequencing. A summary of the variants filtered from known deafness associated genes is presented in Table 2, along with pathogenicity prediction from different in silico bioinformatics tools.

**Table 2:** Summary of results of the massively parallel exome sequencing of two patients with monoallelic mutations in *SLC26A4.* All variants were found in heterozygous state

Patient	Gene	Nucleotide	Protein	dbSNP	Deafness Variation Database	SIFT (score), PolyPhen2 (score), MutationTaster (score), *Splice Prediction Tools*	1000g	ESP6500	Abraom
71	***SLC26A4***	**c.1246A>C**	**T416P**	**rs28939086**	**Pathogenic**	**Damaging (0), Probably Damaging (0.995), Disease causing (0.971541)**	**0.0000**	**0.0000**	**0.0000**
	*GJB2*	c.457G>A	V153I	rs111033186	benign	Tolerated (1), Benign (0.007), Disease causing (0.8156)	0.0013	0.00223	0.0032
	*TMIE*	c.218C>T	T73M	rs770957465	unknown significance	Tolerated (0.156), Probably Damaging (1), Disease causing (0.999)	0.000	0.000	0.000
	***USH1C***	**c.1823C>G**	**P608R**	**rs41282932**	**Pathogenic**	**Damaging (0.002), Probably Damaging (0.984), Disease causing ( 0.9815)**	**0.000**	**0.001**	**0.000**
	*MIR96*	129414574A>G		rs41274239	benign	-	0.0010	0.0033	0.0032
	*PCDH15*	c.4109_4110insGCCGCC	p.P1370delinsPPP	-	not described	-, -. Polymorphism (0.999)	0.0018	0.000	0.0016
	*PCDH15*	c.5134_5136del	p.1712_1712del	rs397517462	not described	-, -. Polymorphism (0.999)	0.000	0.031	0.0032
83	***SLC26A4***	**c.918+2T>C**	**-**	**-**	**Pathogenic**	**-, -, Disease causing (1),** ***splice donor site abolished***	**0.000**	**0.000**	**0.0008**
	***MYO7A***	**c.2463G>C**	**Q821H**	**-**	**not described**	**Damaging (0), Probably Damaging (1), Disease causing (0.9999)**	**0.000**	**0.000**	**0.000**
	***GJB6***	**c.460T>A**	**F154I**	**-**	**not described**	**Damaging (0.022), Damaging (0.986), Disease causing (0.9987)**	**0.000**	**0.000**	**0.000**
	*P2RX2*	c.A275G	p.Q92R	rs142844880	unknown significance	Tolerable (0.116), Benign (0.098), Disease causing (1)	0.0004	0.0004	0.000
	*POLD1*	c.C211T	p.P71S		not described	Tolerable (0.879), Benign (0), non-disease causing (1)	0.000	0.000	0.000
	*TSPEAR*	c.1185G>T	E395D	rs143303485	not described	Tolerated (0.420), Benign (0.02), Polymorphism	0.0002	0.0002	0.000

In bold, variants that all evidence indicate that they are pathogenic

## Discussion

### Pedigrees with autosomal recessive hearing loss

According to the Human Gene Mutation Database [http://www.hgmd.cf.ac.uk/ac/index.php], more than 360 mutations in the *SLC26A4* gene have been identified to date, including splice site aberrations, frameshift, missense and nonsense mutations, as well as large deletions (rare cases) [29]. The mutation spectrum of *SLC26A4* varies widely among ethnic groups, with certain mutations demonstrating a higher prevalence in specific populations [10, 14, 29, 30].

In our study, probably causative mutations in *SLC26A4* were found in 3% (2/68) of the pedigrees presenting autosomal recessive non-syndromic hearing loss. In two of the probands (Patients 6 and 24; Table 1), four different likely pathogenic mutations were found in compound heterozygosis. Three of them were never reported - c.1003 T > G (p. F335 V), c.1553G > A (p.W518X), c.2235 + 2 T > C (IVS19 + 2 T > C), and a fourth had already been described, c.84C > A (p.S28R) [31].

The variant c.1003 T > G is not reported the Deafness Variation Database [24], neither in 1000 Genomes or 6500 Exomes. Computational predictions (Polyphen 2 and Mutation Taster) indicated it as probably disease causing (Table 1). A substitution in the same position, c.1003 T > C (p.F335 L) was reported as causative of Pendred syndrome (Deafness Variation Database) [24], being found with a 0.1% frequency in 1000 Genomes and 6500 exomes, thus, very rare. In addition to the c. 1003 T > G, Patient 6 also carried the c.1553G > A (p.W518X) mutation (compound heterozygosis), unreported in databases, but considered as pathogenic, since it leads to a premature stop codon. A variant affecting the same codon, (c.1554G > A) also leading to a premature stop codon, was described by Pourová et al. [32] as pathogenic. Patient 6 (Table 1) did not show the clinical picture of Pendred syndrome. The same combination of mutations was detected in his affected sister, reinforcing their role as causative of hearing loss.

Patient 24 (Table 1) showed the combination of mutations c.2235 + 2 T > C (IVS19 + 2 T > C) and c.84C > A (p.S28R); c.84C > A (p.S28R), which was already reported in ClinVar as pathogenic, was first described by Fugazzola et al. [33]. It is predicted to be damaging by SIFT, possibly damaging by Polyphen 2, but indicated as a polymorphism by Mutation Taster (Table 1). It is not present in 1000 Genomes and 6500 Exomes. The IVS19 + 2 T > C is a splice site mutation, never reported before. NNSsplice 0.9 version (Lawrence Berkeley National Laboratory, Genomic Informatic Groups, 2012 link) and Netgene2 Server (Center for Biological sequence analysis, 2012 link) predict that the mutation abolishes the splicing donor site of exon 19. After detection of variants c.84C > A and (IVS19 + 2 T > C) in Patient 24, mutation analysis revealed the same mutations in the proband’s affected sister, and that the parents were heterozygous, thus documenting that the mutations were in *trans.* The family was contacted for genetic counseling and recent clinical reassessment indicated that both sisters presented EVA. Given clinical data and segregation data, we concluded that the combination of both mutations explains their phenotype.

Park et al. [10] investigated a collection of Indian and Pakistanese families and selected 15 probands after linkage studies for *SLC26A4* sequencing. Adding two previously reported families [34] to their estimates, the authors came to the conclusion that 17/318 (5%) autosomal recessive cases were attributed to pathogenic variants in the *SLC26A4* gene*.* Following a strategy similar to ours, Pera et al. [35] estimated that 3.5% of pedigrees showing autosomal recessive hearing loss were explained by mutations in *SLC26A4.* The authors investigated 115 pedigrees and selected 20 for mutation screening, after segregation studies with STRs linked to *SLC26A4.*

Albert et al. [31] found that 40% of selected probands for *SLC26A4* screening showed biallelic mutations. However, the selection criteria for analysis included, besides evidence of autosome recessive hearing loss, EVA or other inner ear abnormalities, such as Mondini dysplasia.

Summing up, the finding of 3% of biallelic mutations in our sample, selected on the basis of autosomal recessive hearing loss, is comparable to the studies performed in other populations, with similar criteria of patient selection.

### Patients with EVA or other temporal abnormalities (including Mondini dysplasia) or thyroid malfunctioning

Candidate mutations were found in three out of the 15 patients, (Table 1), but in none of them a second mutation in the same gene was found. In Patient 76, an isolated case born to nonconsanguineous parents, the missense variant c.898C > A (p.I300L) was found in heterozygosis. The variant was previously associated to increased susceptibility to Graves disease (autoimmune hyperthyroidism) [36]. All in silico pathogenicity predictors indicated it as Damaging (Polyphen2–0.02), probably damaging (0.97) and Disease causing (0.99). However, Deafness Variation Database claims it is benign, and databases list this variant, but only in heterozygosis, with a high frequency in healthy African descendent populations (10–18%). There is no phenotype information related to the occurrence of this mutation in homozygosis. Thus, we concluded it is more likely not to be related to the clinical phenotype.

A monoallelic mutation in *SLC26A4,* c.1246A > C, was found in Patient 71, an isolated case of hearing loss, born to non-consanguineous parents. It was already reported as causing Pendred syndrome, and is frequent in Northern European patients with hearing loss [37]. The patient presented bilateral prelingual and progressive hearing loss and computed tomography revealed Mondini dysplasia. After exome sequencing, some variants that could be potentially related to hearing loss were found, but none could individually explain the clinical findings (Table 2).

A monoallelic splice site mutation IVS7 + 2 T > C (c.918 + 2 T > C), listed in the Deafness Variation Database [24] as pathogenic as the cause of non-syndromic hearing loss, and predicted as probably pathogenic by splicing bioinformatic tools, was found in Patient 83, an isolated case, born to non-consanguineous parents. Postlingual progressive hearing loss was observed, but mixed hearing loss was detected in the right ear. MRI indicated EVA. Exome sequencing revealed a set of potentially pathogenic variants in deafness-associated genes, but none could individually explain the hearing loss phenotype (Table 2).

Thus, in 3/15 probands with hearing loss, selected because of EVA or other temporal abnormalities (including Mondini dysplasia) or thyroid malfunctioning, monoallelic mutations were found, but they could not individually explain the clinical findings. In two of them, evidence for pathogenicity of the variants was convincing.

The mutation detection ratio in this sample is smaller than other studies in the literature, in which patients were selected because of clinical signs of Pendred syndrome. However, in most of these studies, patients were selected because they presented hearing loss and EVA and goiter/hypotiroidism. For instance, in the study of Rendtorff et al. [37], 71% of the probands fulfilled at least three diagnostic criteria of Pendred syndrome, which explains the detection rate of 61% of biallelic mutations and 10% of monoallelic mutations in their series. In our sample, only one clinical sign was present in each patient, in addition to hearing loss. The probability of finding *SLC26A4* mutations in this sample is lower, because our criteria of selection were less stringent than most reports in the literature.

De Moraes et al. [38] reported on a *SLC26A4* mutation screening in a selected sample of 23 Brazilian individuals with severe-to-profound non-syndromic hearing loss and EVA. They found 13 different mutations in nine individuals. Five individuals had two mutations (21.7%) and four were found to be monoallelic (17.3%). Although a conclusive molecular diagnosis was possible in many cases, the puzzling high proportion of monoallelic mutations was also seen in their sample.

### MLPA studies

No copy number variation was detected among the patients with only one pathogenic variant (two patients) or among those with no pathogenic allele, detected after Sanger sequencing (27 patients). There are few reports of MLPA technique results regarding the *SLC26A4* gene. Pourová et al. [32] used MLPA to screen for *SLC26A4* deletions and duplications in 18 probands with only one detected mutation, and no copy variation was found. In the study of Zhao et al. [39], in a group of 68 patients with monoallelic mutations and in another group of 39 patients without mutation, no alteration was found. Pique et al. [40], in the investigation of 107 probands with monoallelic mutations in *SLC26A4*, found only one deletion, spanning exons 4–6, which accounted for about 1% of the missing mutations. Pang et al. [41] described a 7666-bp genomic deletion in homozygosis in one patient and in compound heterozygosis in four patients previously classified as monoallelic; this genomic deletion was detected in 18% of the Chinese Han EVA probands with monoallelic *SLC26A4* mutations.

In conclusion, duplications and deletions are rare and do not explain a substantial amount of cases with one mutation or without mutations, after sequencing the *SLC26A4* gene.

### The puzzle of monoallelic mutations

In spite of the well-known recessive nature of mutations in the *SLC26A4* gene, it is striking that, in many reports, a high frequency of individuals presenting only one potentially pathogenic variant is found [37, 42–44].

Pique et al. [40] reviewed six studies [11, 30, 45–48] in which EVA was a selection criteria and came to an estimate that 20% of patients have monoallelic mutations. Two genes, *KCNJ10* [49] and *FOXI1* [48] have been investigated for their role in PDS/DFNB4 disease spectrum and digenic inheritance has been proposed. However, according to Landa et al. [50] and Vona et al. [51], *FOXI1* and *KCNJ10* mutations are rare. In our study, mutations in these two genes were excluded in two of our monoallelic samples, which were submitted to exome sequencing.

Another reasonable explanation for the finding of monoallelic mutations would be the second mutations being deletions or duplications in the *SLC26A4* gene, which, as reasoned above, also seem to be rare in ours and in other studies.

As already stated, we performed exome sequencing in two of the patients with monoallelic mutations (Patients 71 and 83). Although a second mutation was not found in *SLC26A4*, other potentially pathogenic mutations were revealed in other deafness related genes (Table 2), but none could explain deafness from the viewpoint of monogenic inheritance. Of course a quantitative approach is not possible with such a small sample; however, it is tempting to speculate that hearing impairment and related ear malformations in these cases could be due to a multifactorial mechanism. In such a mechanism, a monoallelic *SLC26A4* variant would represent one of the genetic hits needed to phenotype expression. Vona et al. [51] used targeted massive parallel sequencing of a panel of deafness genes in a sample of 30 individuals with hearing loss. About 50% of probands were diagnosed with monogenic forms of nonsyndromic hearing loss, but they found a significant enrichment of potentially pathogenic variants in the undiagnosed affected individuals, when compared to nine hearing controls. Their results, associated with the findings of frequent monoallelic *SLC26A4* mutations in individuals presenting hearing loss and/or EVA, strongly suggest that part of the molecularly undiagnosed cases are due to multifactorial mechanism. Certainly, further studies are needed in the field. This hypothesis could only be verified in larger samples of affected and unaffected individuals submitted to massive parallel sequencing of deafness related genes.

## Conclusions

Our strategy of molecular study of the *SLC26A4* gene allowed the conclusion that biallelic pathogenic mutations in *SLC26A4* explained ~ 3% of cases selected because of autosomal recessive deafness, and that monoallelic mutations were present in ~ 13% of cases of deafness with cochleovestibular malformations or suspected Pendred syndrome. These findings highlight the importance of mutation screening of *SLC26A4* in Brazilian subjects with hearing loss and reinforce the puzzling finding of a high proportion of monoallelic patients, particularly among those presenting cochleovestibular malformations.

## Abbreviations

BWA: Burrows-Wheeler Aligner
DFNB4: Autosomal Recessive Deafness Locus number4
EVA: enlarged vestibular aqueduct
ExAC: Exome Aggregation Consortium
GATK: Genome Analysis Tool Kit
MLPA: Multiplex Ligand Probe Analysis
OMIM: Online Mendelian Inheritance In Man
PS: Pendred syndrome
STR: Short Tandem Repeats
